# Incorporation of a FRET Pair into a Riboswitch RNA to Measure Mg^2+^ Concentration and RNA Conformational Change in Cell

**DOI:** 10.3390/ijms23031493

**Published:** 2022-01-27

**Authors:** Yanyan Xue, Yu Liu

**Affiliations:** State Key Laboratory of Microbial Metabolism, School of Life Science and Biotechnology, Shanghai Jiao Tong University, 800 Dongchuan Road, Shanghai 200240, China; xueyanyan@sjtu.edu.cn

**Keywords:** riboswitch, FRET, in cell, structural dynamics, biosensor

## Abstract

Riboswitches are natural biosensors that can regulate gene expression by sensing small molecules. Knowledge of the structural dynamics of riboswitches is crucial to elucidate their regulatory mechanism and develop RNA biosensors. In this work, we incorporated the fluorophore, Cy3, and its quencher, TQ3, into a full-length adenine riboswitch RNA and its isolated aptamer domain to monitor the dynamics of the RNAs in vitro and in cell. The adenine riboswitch was sensitive to Mg^2+^ concentrations and could be used as a biosensor to measure cellular Mg^2+^ concentrations. Additionally, the TQ3/Cy3-labeled adenine riboswitch yielded a Mg^2+^ concentration that was similar to that measured using a commercial assay kit. Furthermore, the fluorescence response to the adenine of the TQ3/Cy3-labeled riboswitch RNA was applied to determine the proportions of multiple RNA conformational changes in cells. The strategy developed in this work can be used to probe the dynamics of other RNAs in cells and may facilitate the developments of RNA biosensors, drugs and engineering.

## 1. Introduction

Riboswitches are non-coding RNAs located in the 5′-untranslated region of mRNAs and are composed of an aptamer domain and an expression platform. The aptamer domain can differentiate its target ligand from countless metabolites in cells, trigger structural switches, and regulate the transcription or translation of the genes downstream of the riboswitches [1,2]. The functions and applications of riboswitches depend on their precise folding in cells; however, most riboswitch studies have been carried out in vitro [3,4,5,6]. Although in vitro studies are important, their buffered conditions may not reflect the behavior of RNA in more complicated cellular environments because numerous unidentified molecules, concurrent processes, and interactions may contribute to RNA folding and function [7,8]. Therefore, it remains necessary to investigate the natural performance of riboswitches in cells. Chemical modification and hybridization have been routinely used to probe the secondary structures of RNAs in cells [9,10,11,12]. Notably, a recent breakthrough in measuring the populations of RNA conformations in cell was achieved using dimethyl sulfate mutational profiling with the sequencing (DMS-MaPseq) and detection of RNA folding ensembles using expectation maximization (DREEM) [13]. The protein-free regulatory function of riboswitch RNAs relies on their structural flexibility during their binding with specific ligands, and highlights the potential for natural RNA biosensors to detect cellular metabolites [14]. In recent years, spinach, mango, and broccoli aptamers have been coupled to riboswitches to monitor the folding of riboswitch RNA, and have been used as fluorescent light-up aptamers (FLAPs) to detect metabolites in cells [15,16,17,18]. An aptamer-based Förster resonance energy transfer (apa-FRET) system was developed in 2018 to monitor the folding of RNA scaffolds by placing the fluorescent aptamers close to their target RNAs [19]. However, the non-negligible size of an aptamer may disturb the target RNA structure and function [16,20,21].

**Figure 1:**
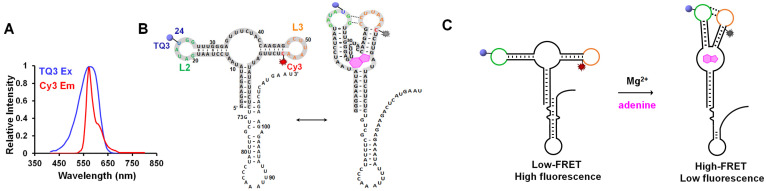
FRET response of TQ3/Cy3-fl upon environmental change. (**A**) Spectral overlap between the emission spectrum of Cy3 (red) with the excitation spectrum of TQ3 (blue). (**B**) The secondary structure of the full-length adenine riboswitch, fl. TQ3 (blue sphere) and Cy3 (red sparkle) are located at the sites 24 and 55, respectively. The aptamer domain, apt, is gray highlighted. (**C**) The structural switch and FRET change of TQ3/Cy3-fl upon Mg^2+^ and adenine. The adenine is shown as magenta polygon.

FRET is widely used to explore the structural dynamics of nucleic acids and proteins. Its efficiency is inversely proportional to the distance between a FRET pair. However, natural RNAs are non-fluorescent; therefore, a donor-acceptor fluorescent pair must be introduced to RNAs to monitor their dynamics using FRET. FRET pairs, such as Cy3–Cy5, Cy5–Cy7, Alexa Fluor 488–555 and Alexa Fluor 555–647, have been used widely in FRET [22,23,24,25,26]. However, a high concentration of acceptors can produce background fluorescence because of crosstalk from donor excitation [27]. Recently, non-fluorescent quenchers have been used to alleviate this problem by releasing the energy from donor to acceptor as heat instead of photons. Donor-quencher pairs Cy3–BHQ (black hole quencher) and Cy3–BHQ2 have been applied to monitor DNA replication and translation processes [27,28]. Tide Quencher 3 (TQ3) is an efficient quencher of Cy3 because they have an excellent spectral overlap (Figure 1A). To our knowledge, the Cy3–TQ3 pair has not been applied to structural studies of RNAs or proteins.

Here, we monitored the folding of an adenine riboswitch using the FRET between Cy3 and TQ3 in vitro and in mammalian cells. A Cy3–TQ3 pair was site-specifically incorporated into the kissing loop (KL) of the full-length adenine riboswitch RNA and its aptamer using position-selective labeling of RNA (PLOR) and a post-synthesis conjugation reaction. PLOR is a solid–liquid hybrid phase transcriptional method, which is capable of incorporating labels into RNAs at desired positions [22,23]. The dynamics of the KL in the full-length riboswitch (TQ3/Cy3-fl) and aptamer (TQ3/Cy3-apt) were probed using FRET in vitro and in cell. We found that the dynamics of the KL in both RNAs were affected more markedly by Mg^2+^ than by adenine, and that adenine affected the dynamics of full-length RNA more than those of the aptamer. The response of the riboswitch to adenine was also weaker and slower in cell than that in vitro. In addition, when used as a Mg^2+^ sensor in mammalian cells, the TQ3/Cy3-labeled adenine riboswitch yielded a Mg^2+^ concentration close to those measured using a commercial assay kit. The TQ3/Cy3-labeled RNA was also used as a structural sensor to measure the proportions of multiple RNA conformational change with adenine.

## 2. Results

### 2.1. Site-Specific Labeling of TQ3/Cy3-apt and TQ3/Cy3-fl

The adenine riboswitch studied in this work was from *Vibrio vulnificus* and can regulate the translation of adenosine deaminase by binding to adenine [4]. The full-length adenine riboswitch, fl, was approximately 110 nt in length and contained the aptamer domain and expression platform (Figure 1B). The aptamer domain, apt (Figure 1B, gray highlighted), was approximately 70 nt in length and contained a kissing loop (G25–C49 and G26–C48) between two hairpins, L2 and L3 (Figure 1B,C and Figure S1) [4,22,29]. The formation of the KL results in high FRET, significant quenching of Cy3 by TQ3, and thus low fluorescence. Conversely, high fluorescence is expected in the absence of the KL (Figure 1C).

**Figure 2:**
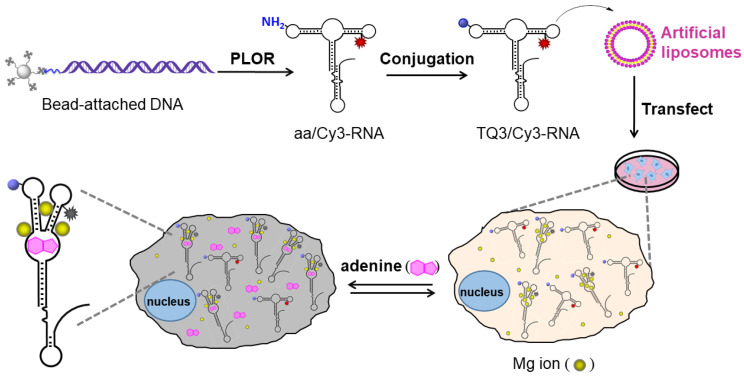
Schematic diagram from the synthesis to cell imaging of TQ3/Cy3-RNA. The Cy3 (red sparkle) and TQ3 (blue sphere) were incorporated into the specific positions of RNA to generate TQ3/Cy3-RNA using PLOR and a conjugation reaction. The TQ3/Cy3-RNA was transfected to tumor cells to monitor its structural dynamics at different environments via live cell imaging. Mg^2+^ and adenine are shown as yellow balls and magenta polygons, respectively.

**Figure 3:**
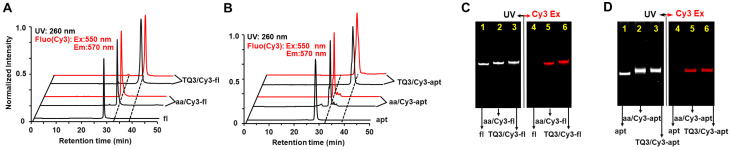
Characterization of aa/Cy3- and TQ3/Cy3-RNA by HPLC and PAGE. HPLC spectra of fl, aa/Cy3-fl and TQ3/Cy3-fl in (**A**) and apt, aa/Cy3-apt and TQ3/Cy3-apt in (**B**) at UV (260 nm, black lines) and fluorescent excitation (550 nm, red lines). PAGE images of fl (lanes 1 and 4), aa/Cy3-fl (lanes 2 and 5) and TQ3/Cy3-fl (lanes 3 and 6) in (**C**) and apt (lanes 1 and 4), aa/Cy3-apt (lanes 2 and 5) and TQ3/Cy3-apt (lanes 3 and 6) in (**D**) at UV (left) and fluorescent irradiation (right).

To monitor the dynamics of the KL in vitro and in cell, we incorporated TQ3 and Cy3 into sites 24 and 55, respectively, for both fl and apt to generate TQ3/Cy3-fl and TQ3/Cy3-apt (Figure 2, Supplementary Tables S1 and S2). Cy3 was introduced to site 55 of the two RNAs using the PLOR method. The direct introduction of TQ3 to an RNA by PLOR was impracticable because TQ3-labeled NTPs are not commercially available. Therefore, we used PLOR to incorporate an aminoallyl (aa) nucleotide at site 24, and the resulting aa/Cy3-fl and aa/Cy3-apt were then labeled using an amine-reactive N-hydroxysuccinimide ester of TQ3 (NHS-TQ3) (Figure S2). In the RP-HPLC, TQ3/Cy3-fl and TQ3/Cy3-apt eluted from a C8 column later than their aa/Cy3-labeled counterparts, and aa/Cy3-fl and aa/Cy3-apt eluted later than the corresponding unlabeled fl and apt RNAs (Figure 3A,B). In the 12% denaturing PAGE, aa/Cy3- and TQ3/Cy3-labeled RNAs migrated under UV irradiation slightly slower than their unlabeled counterparts (Figure 3C,D). Additionally, only aa/Cy3- and TQ3/Cy3-labeled RNAs were visible under fluorescence excitation during both HLPC (red curves, Figure 3A,B) and PAGE (red bands, Figure 3C,D). A longer elution time in HPLC, a slower migration in PAGE and fluorescent visibility were attributed to the hydrophobic, bulky and fluorescent nature of Cy3 and/or TQ3, and provided evidence for the successful introduction of Cy3 and/or TQ3 to the RNAs. In addition, the single peak (band) in the HPLC and PAGE indicated the high purity of the labeled RNAs.

### 2.2. Influence of Mg^2+^ and Adenine on the Structural Dynamics of TQ3/Cy3-apt and TQ3/Cy3-fl In Vitro

**Figure 4:**
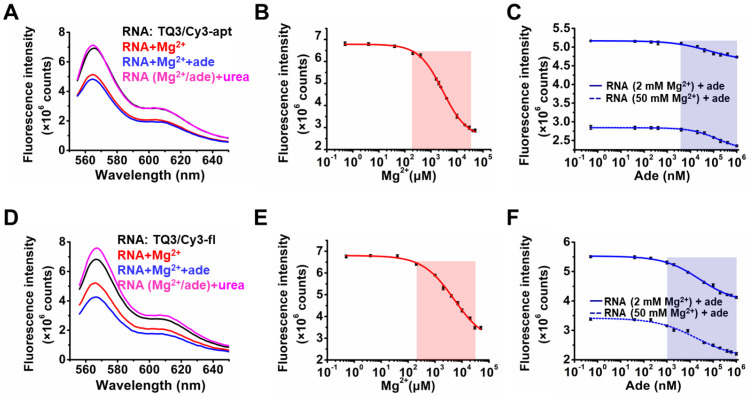
Steady-state fluorescence of TQ3/Cy3-apt and TQ3/Cy3-fl at different buffer conditions in vitro. (**A**) Fluorescence spectra of TQ3/Cy3-apt (black), with 2 mM Mg^2+^ (red), 2 mM Mg^2+^ and 0.1 mM adenine (blue) and 6 M urea (magenta). (**B**) Titration curves of 0.5 μM–50 mM Mg^2+^ to TQ3/Cy3-apt. (**C**) Titration curves of 0.5 nM–1 mM adenine to TQ3/Cy3-apt in the presence of 2 mM Mg^2+^ (solid) or 50 mM Mg^2+^ (dotted). (**D**) Fluorescence spectra of TQ3/Cy3-fl (black), with 2 mM Mg^2+^ (red), 2 mM Mg^2+^ and 0.1 mM adenine (blue) and 6 M urea (magenta). (**E**) Titration curves of 0.5 μM–50 mM Mg^2+^ to TQ3/Cy3-fl. (**F**) Titration curves of 0.5 nM–1 mM adenine to TQ3/Cy3-fl in the presence of 2 mM Mg^2+^ (solid) or 50 mM Mg^2+^ (dotted). Fluorescence intensity was replicated 3 times, shown as means ± standard deviations. The deviations were less than 2%.

We first measured the responses of TQ3/Cy3-fl and TQ3/Cy3-apt in the buffer of 10 mM HEPES, 100 mM KCl, pH 7.5 to Mg^2+^ and adenine in vitro. The steady-state fluorescence of TQ3/Cy3-apt decreased by approximately 25% upon the addition of 2 mM Mg^2+^, and decreased a further 5% upon the subsequent addition of 0.1 mM adenine (Figure 4A), indicating that Mg^2+^, rather than adenine, contributes to KL formation in the aptamer domain, as reported elsewhere [30]. A fluorescence recovery was observed upon the subsequent addition of 6 M urea, which indicated that KL barely formed in the absence of Mg^2+^ (Figure 4A). To further clarify the conformational change of TQ3/Cy3-apt in response to Mg^2+^ and adenine binding, 0.5 μM–50 mM Mg^2+^ was titrated to the RNA. The fluorescence of TQ3/Cy3-apt markedly decreased by approximately 63% upon the addition of 50 mM Mg^2+^ (Figure 4B), with the calculated *K_D_* of 2.87 mM being comparable with previously reported data (Supplementary Table S3) [31]. In contrast to Mg^2+^, the impact of adenine on the KL was much less pronounced. Then, 0.5 nM–1 mM adenine was titrated to TQ3/Cy3-apt (premixed with 2 or 50 mM Mg^2+^), the fluorescence barely changed until adenine reached 4 μM (Figure 4C). As the concentrations of adenine were within 4 μM–1 mM, the fluorescence kept decreasing with adenine addition. Significantly, 1 mM adenine decreased the fluorescence of TQ3/Cy3-apt by approximately 8% and 17% in the presence of 2 and 50 mM Mg^2+^, with *K_D_* values of 61.83 and 66.55 μM at 2 and 50 mM Mg^2+^, respectively (Figure 4C and Supplementary Table S3). These in vitro results suggest that Mg^2+^, rather than adenine, plays a critical role in KL formation, and that the KL in the aptamer is sensitive to Mg^2+^ and adenine in concentration ranges of 0.2–30 mM and 4 μM–1 mM, respectively (Figure 4B,C).

The fluorescence of TQ3/Cy3-fl decreased by approximately 25% and 49% upon the addition of 2 and 50 mM Mg^2+^, respectively (Figure 4D,E), and similar results were observed for TQ3/Cy3-apt, indicating that both RNAs had Mg^2+^-dependent KL formation. Moreover, 0.5 nM–1 mM adenine were titrated to TQ3/Cy3-fl in the presence of 2 or 50 mM Mg^2+^, and the fluorescence decreased with the addition of 1 μM–1 mM adenine (Figure 4F). The addition of 1 mM adenine reduced the fluorescence of TQ3/Cy3-fl by approximately 25% and 33% in the presence of 2 and 50 mM Mg^2+^, respectively, which suggested that adenine binding affected the dynamics of the full-length RNA more markedly than those of its aptamer domain alone (Figure 4F). The *K_D_* value for Mg^2+^ binding to TQ3/Cy3-fl was 3.99 mM, and the corresponding values for adenine binding were 15.83 and 13.23 μM at 2 and 50 mM Mg^2+^, respectively (Supplementary Table S3). The full-length RNA bound more weakly than its aptamer to Mg^2+^ but more strongly than the aptamer to adenine (Supplementary Table S3). These results indicate that both Mg^2+^ and adenine are crucial for KL formation in the full-length adenine riboswitch. Moreover, urea increased the fluorescence of TQ3/Cy3-fl more than that of TQ3/Cy3-apt, indicating that more full-length RNA pre-folded with the KL in the absence of Mg^2+^ and adenine than did its aptamer (Figure 4A,D). Taken together, these results suggest that the full-length adenine riboswitch was more stable in the absence of Mg^2+^ and adenine and exhibited more pronounced adenine-dependent structural transitions than the aptamer. In addition, the KL in the full-length RNA was sensitive to Mg^2+^ and adenine in concentration ranges of 0.2–30 mM and 1 μM–1 mM, respectively, in vitro (Figure 4E,F). As negative controls, the fluorescence of aa/Cy3-apt and aa/Cy3-fl changed negligibly following the addition of Mg^2+^ and adenine (Figure S4).

### 2.3. Application of TQ3/Cy3-RNA as a Biosensor of Cellular Mg^2+^ Concentration

**Figure 5:**
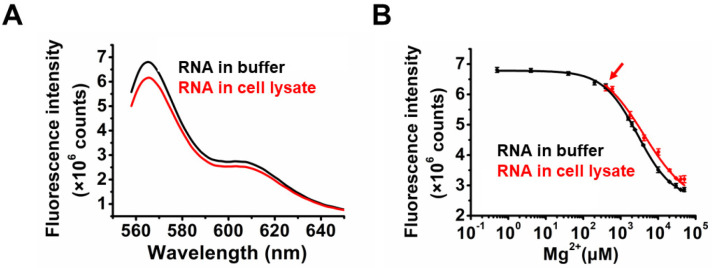
Cellular Mg^2+^ concentration detection using TQ3/Cy3-RNA. (**A**) Fluorescence spectra of TQ3/Cy3-apt in the buffer (10 mM HEPES, 100 mM KCl, pH 7.5, black) and in the A549 cell lysate (red). (**B**) Titration curves of Mg^2+^ to TQ3/Cy3-apt in the buffer (10 mM HEPES, 100 mM KCl, pH 7.5, black) and in the A549 cell lysate (red). Red arrow points to the starting titration (without extra addition of Mg^2+^), corresponding to approximately 0.4 mM Mg^2+^ in the cell lysate. Each spectrum was replicated 3 times.

Given the marked Mg^2+^-dependent KL formation of TQ3/Cy3-RNA in response to 0.2–30 mM Mg^2+^ in vitro, we used this RNA as a biosensor of cellular Mg^2+^ concentration. A decrease in fluorescence of 10.8% was observed after TQ3/Cy3-RNA was added to a lysate of A549 cells (Figure 5A), consistent with the change induced by 0.4 mM Mg^2+^ in vitro (Figure 5B, red arrow). Accounting for the volume change accompanying cell lysis, the Mg^2+^ concentration in live A549 cells was adjusted to approximately 0.63 mM, which is comparable with previously reported data [32]. We also added 0.2–50 mM Mg^2+^ to the A549 cell lysate and observed a Mg^2+^-related fluorescence decrease (Figure 5B, red line). The titration curve of TQ3/Cy3-RNA binding to Mg^2+^ in cell lysate was similar to that obtained in buffer (Figure 5B). Consistent with our findings, Mg^2+^ concentrations of 0.5–0.7 mM were measured in the cell lysate using the commercial cell Mg^2+^ assay kit from GenMed Scientifics.

### 2.4. Conformational Change of TQ3/Cy3-apt and TQ3/Cy3-fl in Mammalian Cells

**Figure 6:**
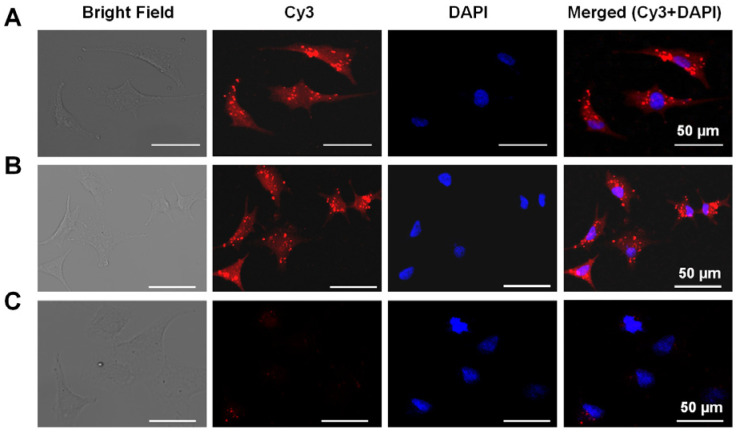
Confocal microscopic images of the fixed A549 cells after being transfected with TQ3/Cy3-apt (**A**) TQ3/Cy3-fl (**B**) and free Cy3 dye. (**C**). Red and blue fluorescence spots were from Cy3 and DAPI, respectively.

**Figure 7:**
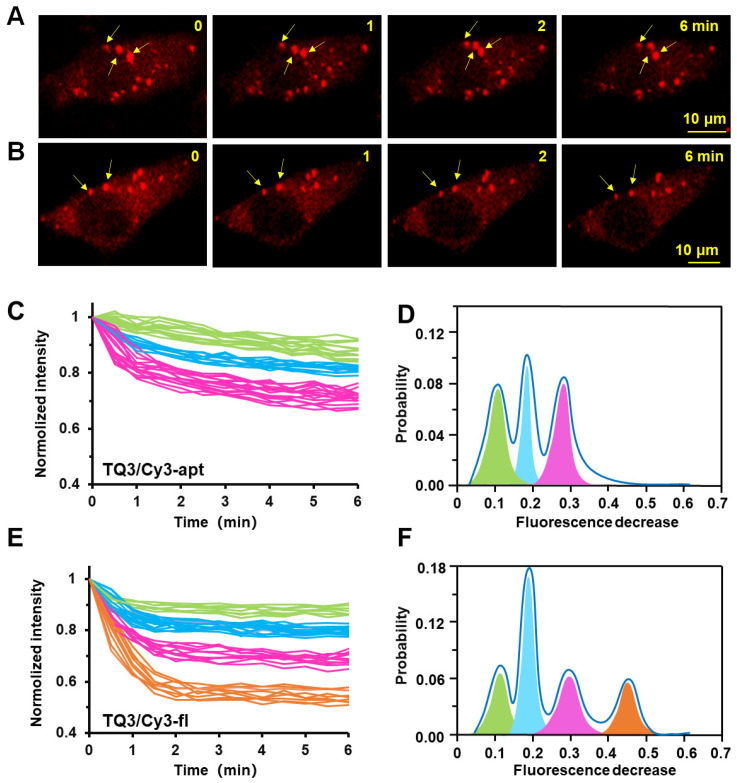
Live cell imaging and conformational switches of TQ3/Cy3-apt and TQ3/Cy3-fl upon the addition of adenine. Time course confocal imaging of TQ3/Cy3-apt (**A**) and TQ3/Cy3-fl (**B**) in live A549 cells upon the adenine addition. The fluorescence decrease in multiple TQ3/Cy3-apt foci (**C**) and TQ3/Cy3-fl foci (**E**) in live cells measured in 30 s intervals following being induced by adenine. Histogram of conformational switches at TQ3/Cy3-apt (**D**) and TQ3/Cy3-fl (**F**) in live cells at 6 min following adenine addition.

To monitor the adenine-induced changes in the structural dynamics of TQ3/Cy3-fl and TQ3/Cy3-apt in cell, we transferred the RNAs to A549 tumor cells using liposome-mediated transfection (Figure 2). Confocal images showed that both TQ3/Cy3-apt and TQ3/Cy3-fl formed discrete particles in the cytoplasm (Figure 6A, B). As a negative control, the free Cy3 dye was minimally transfected into cells and created negligible background fluorescence (Figure 6C). Time-series confocal imaging showed that both TQ3/Cy3-apt and TQ3/Cy3-fl displayed an adenine-dependent conformation switch with time in live A549 cells. We observed that TQ3/Cy3-RNA were markedly less sensitive to adenine in cell than in vitro, which was supported by the fact that a clear reduction of TQ3/Cy3-apt and TQ3/Cy3-fl fluorescence was observed with the addition of 1 mM or higher of adenine, after testing with 1 μM–10 mM adenine (Figure 7A,B). However, there was no distinct fluorescence difference between 1 mM and 10 mM adenine additions. Moreover, the response of the RNAs to adenine was slower in cell than in vitro, with the fluorescence of TQ3/Cy3-apt and TQ3/Cy3-fl reaching a steady state in less than 1 min in the latter case, but taking approximately 6 min in the former (Figure 7A,B). After 6 min, the fluorescent intensity of TQ3/Cy3-RNA barely altered with time. This may be because the crowed cellular environment impedes the access of adenine to the RNAs. The fluorescence of aa/Cy3-apt and aa/Cy3-fl changed negligibly upon adenine addition in cell, indicating that the decreases observed for TQ3/Cy3-apt and TQ3/Cy3-fl were the result of conformational changes and not because of the photo-bleaching of Cy3 (Figure S5A,B).

Although TQ3/Cy3-apt and TQ3/Cy3-fl were less sensitive to adenine in cell than in vitro, the fluorescence change of individual RNA foci could be quantified over time. At low concentrations, the RNA molecules were distributed as discrete particles, foci in the cells. The fluorescence intensities of more than 100 RNA foci (Figure 7A,B, yellow arrow) from different cells were measured in 30 s intervals up to 6 min post adenine addition (Figure 7C,E). The fluorescence of individual foci was measured at least three times, with a deviation of less than 1.5%. The fluorescence decrease in TQ3/Cy3-apt and TQ3/Cy3-fl up to 6 min after adenine addition was fitted to Gaussian functions (Figure 7D,F). Compared with the ensemble results in vitro, the differences between TQ3/Cy3-apt and TQ3/Cy3-fl were better resolved in cell. The initial fluorescence of RNA foci was normalized, and those with a difference of less than 10% at the final fluorescence were grouped into one group. The fluorescence decreases could be divided into three and four groups for the aptamer and full-length RNA, respectively (Figure 7C–F). For TQ3/Cy3-apt, adenine-induced changes in KL formation were classified as slight (0.10, green), moderate (0.18, cyan) or distinct changes (0.28, magenta) (Figure 7D). In contrast, the corresponding changes for TQ3/Cy3-fl were more marked, and were classified as slight (0.12, green), moderate (0.18, cyan) distinct (0.30, magenta) or drastic changes (0.45, orange) (Figure 7F). The corresponding changes for the aa/Cy3-apt and aa/Cy3-fl were negligible (Figure S5C,D). Similar to the in vitro findings, the structural switch in the full-length RNA was more sensitive than the isolated aptamer domain to adenine in cell. Our results suggest that TQ3/Cy3-RNAs can be used to quantify the relative proportions of multiple coexisting conformation changes in cell.

## 3. Discussion

The investigation of the structural dynamics of cellular RNAs is essential to understand their functions and to develop RNA-based drugs and biosensors. Here, we incorporated a FRET pair into an adenine riboswitch RNA and its aptamer using PLOR and a conjugation reaction. The FRET pair was used to monitor conformations of the RNAs and measure Mg^2+^ concentrations in cell. To our knowledge, the Cy3–TQ3 FRET pair has not previously been used in structural studies of RNAs. The in vitro KL formation in full-length RNA and its aptamer depended markedly on Mg^2+^ concentrations, but weakly on adenine concentrations. We also tested the effects of monovalent and other divalent metal ions in vitro, and the fluorescence of TQ3/Cy3-apt decreased negligibly even when the Na^+^ or K^+^ levels reached as high as 100 mM (Figure S6). On the contrary, the TQ3/Cy3-apt was sensitive to other divalent metal ions, such as Ca^2+^ and Mn^2+^. With the addition of 0.5 μM–50 mM Ca^2+^ or Mn^2+^, the fluorescence of TQ3/Cy3-apt decreased in the range of 0.2–50 mM for both Ca^2+^ or Mn^2+^, and the calculated *K_D_* of Ca^2+^ or Mn^2+^ was comparable with that of Mg^2+^ (Figure S7). However, the cellular concentrations of Ca^2+^ or Mn^2+^ are much lower than Mg^2+^ [32,33,34]; therefore, the effects of Ca^2+^ and Mn^2+^ on the fluorescence are much less significant than Mg^2+^ in cell. Additionally, the response of the adenine riboswitch to adenine is highly specific, supported by the insensitive response of the RNA to adenine analogs, ATP and guanine (Figure S8). The influence of adenine on KL formation in vitro was greater for the full-length RNA than for the aptamer. A comparable influence of adenine on the conformational switching was observed in cell using confocal microscopy. Consistent with the in vitro findings, the TQ3/Cy3-labeled RNAs were insensitive to the cellular adenine, but could be used to measure the relative proportions of multiple RNA conformational change with adenine in cell, which is usually challenging. The strategy developed in this work has the potential to be developed as a general and convenient tool for probing other RNAs in cell, such as regulatory viral RNAs, and to contribute to biosensor development and disease diagnosis.

## 4. Materials and Methods

### 4.1. Preparation of DNA Templates for PLOR Reactions

The DNA templates of PLOR reactions were produced via PCR with biotin and 2′-O-methyl groups at the 5′-ends of the coding and non-coding strand, respectively [22,23]. The sequences of the DNAs used in the PCR reactions are listed in Supplementary Table S1. The biotin was used to immobilize the DNA templates on the streptavidin-coated agarose beads, which allowed PLOR to conveniently exchange reagents and buffers by filtration. Then, 2′-O-methyl was used to reduce the unwanted non-template transcripts. The DNA templates were purified by 12% urea-PAGE, and then incubated with the streptavidin-coated agarose beads (Smart-Life sciences, Changzhou, China) at 4 °C overnight to immobilize the DNA to the beads, generating solid-phase DNA-beads for use as the DNA templates in PLOR reactions. The immobilized DNA-beads were stored at 4 °C for future usage.

### 4.2. PLOR Generation of aa/Cy3-apt and aa/Cy3-fl

PLOR were used to synthesize aa/Cy3-apt and aa/Cy3-fl (aa and Cy3 at sites 24 and 55). Various conditions, including concentrations of DNA-beads, T7 RNA polymerase (RNAP), aa-UTP, Cy3-CTP and reaction temperatures for Cy3-CTP incorporation, were optimized for the synthesis of aa/Cy3-labeled RNA by PLOR. Then, 5, 10, 15 and 20 μM DNA with equal T7 RNAP were tested, and the efficiency was optimized at 15 μM (Figure S3A). Different ratios (0.5:1, 0.8:1, 1:1 and 1.5:1) of T7 RNAP to DNA were tested, and the highest efficiencies were observed for 1:1 and 1.5:1 (Figure S3B). Following this, 25, 30 and 37 °C were screened at step 10 to improve the tolerance of T7 RNAP for the bulky Cy3-CTP, and the results indicated that 30 °C behaved slightly better (Figure S3C). In short summary, the optimal conditions of PLOR for synthesizing aa/Cy3-RNA were a 15 μM DNA template, an equal molar concentration of T7 RNAP, and 30 °C at step 10.

The synthesis procedure for the aa/Cy3-fl was divided into 11 steps (Figure S2A), and the NTP additions at individual step are listed in Supplementary Table S2. At the 1st step (initiation stage), 15 μM T7 RNAP and DNA-beads, 1.44 mM ATP, 0.96 mM GTP and 144 μM UTP were incubated at 37 °C for 15 min, producing the 13 nt RNA fragment. Filtration and bead-rinsing were performed 3 times to remove the residual NTPs from the reaction container. The product extensions in steps 2 to 9 were performed at 25 °C for 10 min, followed by filtration and bead-rinsing. The chemically modified nucleotides, aa-UTP (TriLink Biotechnologies, San Diego, CA, USA) and Cy3-CTP (GE Healthcare, Chicago, IL, USA), were incorporated into the nascent transcribed RNA at steps 5 and 10, respectively. To improve the incorporation efficiency of T7 RNAP for the bulky Cy3, step 10 was performed at 30 °C. After labeling sites 24 and 55, four types of NTPs were added to complete the synthesis of aa/Cy3-fl at 25 °C for 10 min at step 11, producing more than 6 moles of aa/Cy3-apt in a 2 mL reaction. The procedure of PLOR for aa/Cy3-apt synthesis was the same as aa/Cy3-fl, except for the DNA templates and the NTP additions at step 11 (Supplementary Table S2). Both aa/Cy3-apt and aa/Cy3-fl were purified using 12% urea-PAGE, exchanged into DEPC-H2O and lyophilized for future usage.

### 4.3. Post-Synthesis Incorporation of TQ3 into aa/Cy3-apt and aa/Cy3-fl

For this process, 3.3 μL of 2 mM aa/Cy3-labeled RNA, 2 μL of 1 M NHS-TQ3 (AAT Bioquest, Sunnyvale, CA, USA), 3 μL DMSO and 1.7 μL of 0.3 M NaHCO_3_ (pH 8.4) were shaken for 3 h at room temperature in the darkness to generate TQ3/Cy3-labeled RNA [35]. The products, TQ3/Cy3-apt and TQ3/Cy3-fl, were purified by 12% urea-PAGE to remove the residual free NHS-TQ3 and other reagents. Then, TQ3/Cy3-apt and TQ3/Cy3-fl were purified by RP-HPLC using a C8 column via the following procedure: the first 2 min was with 10% buffer B (75% acetonitrile with 100 mM TEAA) and 90% buffer A (DEPC-H_2_O with 100 mM TEAA), which was then ramped from 10 to 70% buffer B over the next 40 min (flow rates are 0.3 mL/min). The desired product was collected under a fluorescent detector of HPLC, and exchanged to the desired buffer for further experiments. The purity of TQ3/Cy3-apt and TQ3/Cy3-fl was checked using urea-PAGE under UV irradiation (302 nm) or with a ChemiScope instrument (fluorescent excitation of wavelength 530 nm) (CLiNX, Shanghai, China). Except those noted, all RNA samples were refolded by heating at 85 °C for 5 min and then cooling down to room temperature before usage.

### 4.4. Steady-State Fluorescence Detection

The steady-state fluorescence spectra (550~650 nm) of 0.4 μM, 100 μL RNA samples in the buffer (10 mM HEPES, 100 mM KCl, pH 7.5) titrated with 0.5 μM–50 mM Mg^2+^ MgCl_2_ were collected with a FLS1000 photoluminescence spectrometer (Edinburgh Instruments Ltd., Edinburgh, UK). Then, 0.4 μM, 100 μL RNA samples in the buffer (10 mM HEPES, 100 mM KCl, 2 or 50 mM MgCl_2_, pH 7.5) were titrated with 0.5 nM–1 mM adenine. The volume change caused by MgCl_2_ or adenine titration in an individual experiment was less than 5% of the initial volume. During measurement, the excitation wavelength was set at 550 nm, with a band width of 0.3 nm, and the emission signal was collected at 1 nm/s from wavelength 550 to 650 nm, with a band width of 7 nm. The fluorescence measurements were repeated five times and their averages were plotted. All data were fit using a sigmoidal model in Origin software (OriginLab, MA, USA) to obtain the apparent dissociation constant (*K_D_*) of Mg^2+^ or adenine binding with RNA (Supplementary Table S3).

### 4.5. Cell Culture and RNA Transfection

A549 cells were cultured in Dulbecco’s Modified Eagle medium (DMEM, Gibco Life Technologies, Grand Island, NY, USA), containing 10% fetal bovine serum (Gibco Life Technologies) and 1% penicillin–streptomycin (Gibco Life Technologies) at 37 °C in humidified air with 5% CO_2_. For fixed-cell imaging, the cells were seeded on glass coverslips in a 12-well plate and grown for 1 day to approximately 30% confluence before transfection. For live cell imaging, the cells were seeded in the specialized confocal imaging cell culture dish (NEST, Wuxi, China) and grown for 1 day to approximately 70% confluence prior to transfection. Cells were transfected with Cy3 dyes or RNA samples (10 pmol each) using the Lipofectamine MessengerMax Reagent (Invitrogen, Carlsbad, CA, USA), following the manufacturer’s instruction. After incubation at 37 °C for 4 h, the cells were washed five times with phosphate-buffered saline (PBS) to remove the residual dye or RNA, then fixed in 4% paraformaldehyde (Invitrogen) at room temperature for 30 min. The fixed cells were washed three times with PBS to remove the paraformaldehyde. To visualize their nuclei, the cells were soaked in 5 μg/mL DAPI at room temperature for 10 min. Excess DAPI was removed by washing the cells with PBS three times. The coverslip was then placed on a microscope slide containing 20 μL antifade mountant (Invitrogen) for subsequent confocal imaging.

### 4.6. Cell Imaging Using Confocal Microscopy

DAPI-stained cells transfected with aa/Cy3-apt, TQ3/Cy3-fl, aa/Cy3-fl or TQ3/Cy3-fl were examined using a Leica SP8 STED confocal inverted microscope equipped with a 20× or 40× emission objective lens (Leica, HE, Gremany). Excitation wavelengths of 405 and 514 nm was used for imaging DAPI and Cy3, respectively. The emission spectra of DAPI and Cy3 were measured in the 425–475 and 560–600 nm ranges, respectively. Confocal images of DAPI-stained cells were obtained in three-dimensional x, y, z mode using 0.3 μm increments along the *z*-axis.

Time series confocal imaging was used to measure changes in RNA fluorescence in live cells. Adenine was added to a working concentration of approximately 1 mM in the culture medium of the transfected cells. Confocal images were obtained at 37 °C every 30 s for 15 min after adenine addition. During the first 6 min, the fluorescence of TQ3/Cy3-apt and TQ3/Cy3-fl gradually decreased. After 6 min, it was minimally affected. The time-dependent fluorescence intensities of more than 100 random RNA foci in individual cells were quantified using the Image J software by measuring the fluorescence in a region of interest (ROI) divided by the ROI area (mm^2^) in 30 s intervals up to 6 min post adenine addition, and the intensities of individual foci were normalized to their value at 0 min. In addition, the total fluorescence decrease between 0 and 6 min was calculated, and the distributions of the obtained values were fitted to a sum of Gaussian functions using the Origin software.

### 4.7. Measurement of Cellular Mg^2+^ Concentration

A549 cells were cultured on a 10 cm diameter dish until the cell monolayer was overgrown. The cells were then washed three times with PBS and collected after digestion with 0.25% trypsin-EDTA (Gibco Life Technologies). The cell suspension was lysed on ice for 40 min in PBS containing 0.5% Triton X-100, then centrifuged to obtain the lysate. TQ3/Cy3-apt was diluted in the cell lysate to a working concentration of 0.4 μM, and steady-state fluorescence measurements of the TQ3/Cy3-apt fluorescence were conducted as described above. The fluorescence intensity of TQ3/Cy3-apt in buffer (10 mM HEPES, 100 mM KCl, pH 7.5) was compared with that of the cell lysate to deduce the Mg^2+^ concentration in the lysate. The cellular Mg^2+^ concentration was then calculated using the following equation:

$$C_{C}=\frac{C_{L}*V_{L}}{V_{C}}$$

where C_C_ is the live cell Mg^2+^ concentration, V_c_ is the cell volume, $C_{L}$ is the lysate Mg^2+^ concentration and V_L_ is the lysate volume. The live cell volume was calculated as the product of the culture surface area and the average thickness of the A549 cell monolayer. In this study, V_C_ and V_L_ were approximately 120 μL and 190 μL, respectively.

Following this, 0.2–50 mM Mg^2+^ were titrated to the cell lysate, then the fluorescence intensity was fitted using the sigmoidal model in the Origin software to obtain the apparent dissociation constant, *K_D_*, for Mg^2+^ and TQ3/Cy3-apt. The cell lysate Mg^2+^ concentration was also measured using a commercial assay kit, following the manufacturer’s instructions (Genmed Scientifics Inc., Arlington, MA, USA).

## Data Availability

The data presented in this study are available in insert article or Supplementary Material here.

## Supplementary Materials

The following supporting information can be downloaded at: www.mdpi.com/article/10.3390/ijms23031493/s1.
