# Supplementary material for: Incorporation of a FRET Pair into a Riboswitch RNA to Measure Mg2+ Concentration and RNA Conformational Change in Cell
Source: Int J Mol Sci. 2022 Jan 27;23(3):1493. doi: 10.3390/ijms23031493 (PMC8835884; doi:10.3390/ijms23031493)
Supplement: Supplementary file 1 [file ijms-23-01493-s001.zip › ijms-1528440-supplementary-done.pdf]

International Journal of
Molecular Sciences

MDPI

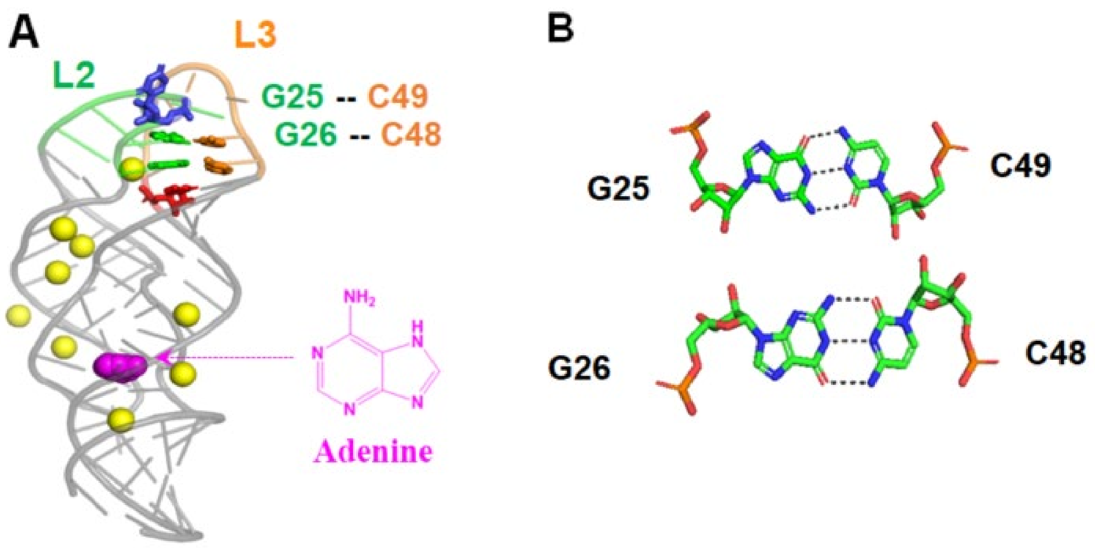

Supplementary Figure S1. (A) The crystal structure of the aptamer domain of adenine riboswitch at the presence of adenine (PDB ID: 4TZX). A kissing loop (KL) with two base-pairs, G25-C49 and G26-C48 is formed between L2 (green) and L3 (orange). (B) The hydrogen bonds of G25-C49 and G26-C48. Base pairings are indicated with dashed lines. The carbon, nitrogen, oxygen and phosphate atoms are colored in green, blue, red and orange, respectively.

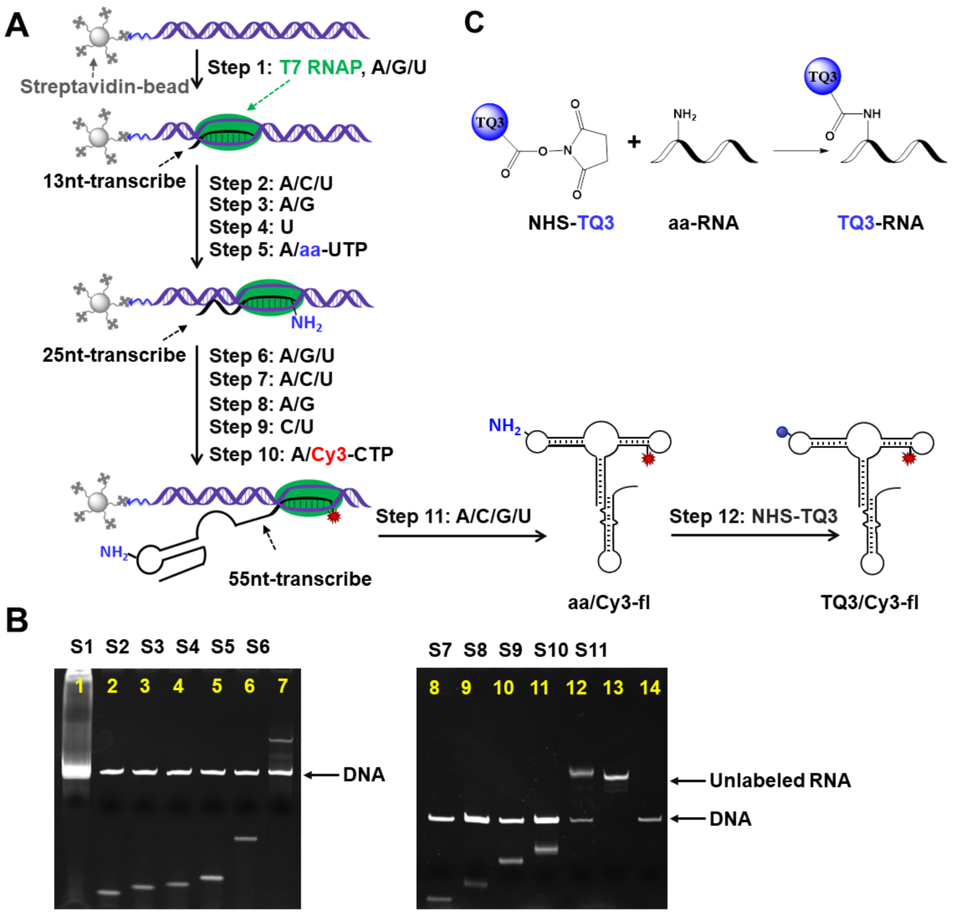

Supplementary Figure S2. Diagram of aa/Cy3 and TQ3/Cy3-fl synthesis by PLOR and a conjugation reaction. (A) PLOR reaction (steps 1-11) was applied to produce aa/Cy3-fl, which was specifically labeled with aa and Cy3 group at sites 24 and 55, respectively. The conjugation reaction (step 12) between aa/Cy3-fl and NHS-TQ3 was performed after PLOR to generate TQ3/Cy3-fl. aa, Cy3 and TQ3 groups are shown as NH_2 (blue), sparkle (red), and sphere (blue), respectively. The biotin-labeled double-stranded DNA (purple) was attached to the streptavidin-coated agarose beads (gray) and used as the solid-phase DNA template in PLOR. (B) 12% denaturing PAGE of products at individual step in PLOR. The eluents at steps 1 to 11 were loaded at lanes 1-6 and 8-12. The double-stranded DNA templates were loaded at lanes 7 and 14, and unlabeled full-length RNA was loaded at lane 13 as standard samples. (C) The conjugation reaction between aa-RNA and NHS-TQ3 to obtain the TQ3-RNA.

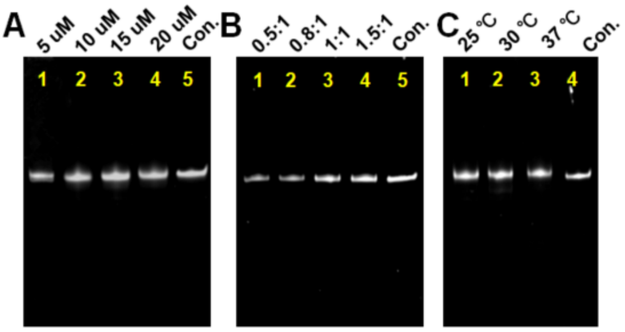

Supplementary Figure S3. Optimization of PLOR synthesis for aa/Cy3-fl. (A) The DNA concentrations are 5 (Lane 1), 10 (Lane 2), 15 (Lane 3) and 20 µM (Lane 4), respectively. Unlabeled fl was loaded at Lane 5 as control. (B) Optimization of the ratios between T7 RNAP and DNA template. The ratios are 0.5:1 (Lane 1), 0.8:1 (Lane 2), 1:1 (Lane 3), and 1.5:1 (Lane 4). Unlabeled fl was loaded at Lane 5 as control. (C) Optimization of reaction temperatures at step 10 for Cy3-CTP incorporation. The reaction temperatures are 25 ℃e(Lane 1), 30 ℃ (Lane 2), and 37 ℃ (Lane 3). Unlabeled fl was loaded at Lane 4 as control.

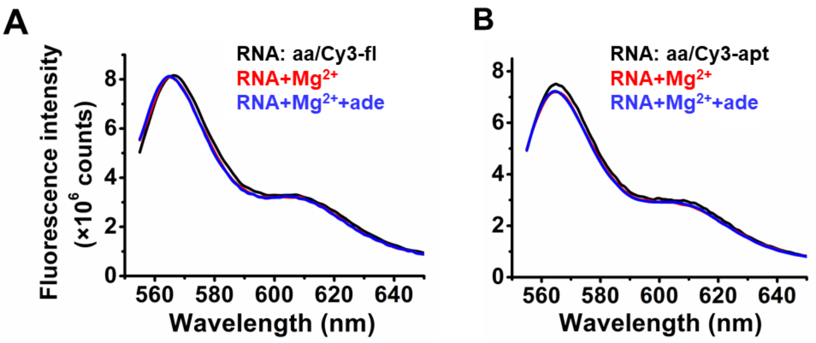

Supplementary Figure S4. Steady state fluorescence detection of aa/Cy3-fl and apt response to Mg^{2+} and adenine. (A) Fluorescence spectra of aa/Cy3-fl (black), aa/Cy3-fl with 2 mM Mg^{2+} (red) and aa/Cy3-fl with 0.1 mM adenine (blue). The 0.1 mM adenine was titrated to the RNA premixed with 2mM Mg^{2+} (blue). (B) Fluorescence spectra of aa/Cy3-apt (black), aa/Cy3-apt with 2 mM Mg^{2+} (red) and aa/Cy3-apt with 0.1 mM adenine (blue). The 0.1 mM adenine was titrated to the RNA premixed with 2mM Mg^{2+} (blue). Each spectrum was replicated 3 times, fluorescence intensity values were means ± standard deviations of triplicate experiments.

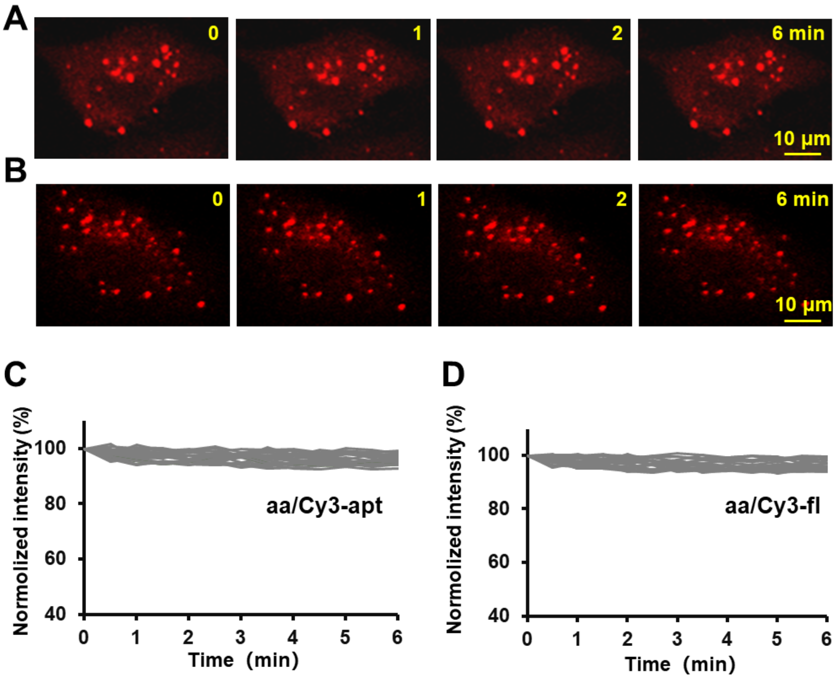

Supplementary Figure S5. Cellular conformational distribution of aa/Cy3-apt and fl based on fluorescence decrease induced by adenine. Time course confocal imaging of aa/Cy3-apt (A) or aa/Cy3-fl (B) in live A549 cell after the adenine addition. Time course of fluorescence decrease of multiple aa/Cy3-apt foci (C) or aa/Cy3-fl foci (D) in live cells induced by adenine. The fluorescence intensities of RNA foci from different cells were measured in 30 s intervals up to 6 min post adenine addition.

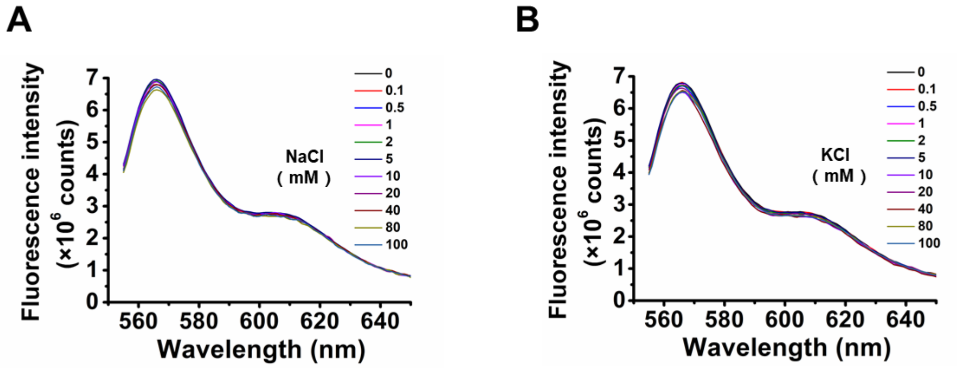

Supplementary Figure S6. Steady-state fluorescence response of TQ3/Cy3-apt to monovalent metal ions. (A) Steady-state fluorescence spectra of TQ3/Cy3-apt with the addition of 0.1–100 mM Na^+. (B) Steady-state fluorescence spectra of TQ3/Cy3-fl with the addition of 0.1–100 mM K^+. Each spectrum was replicated 3 times, fluorescence intensity values were means ± standard deviations of triplicate experiments.

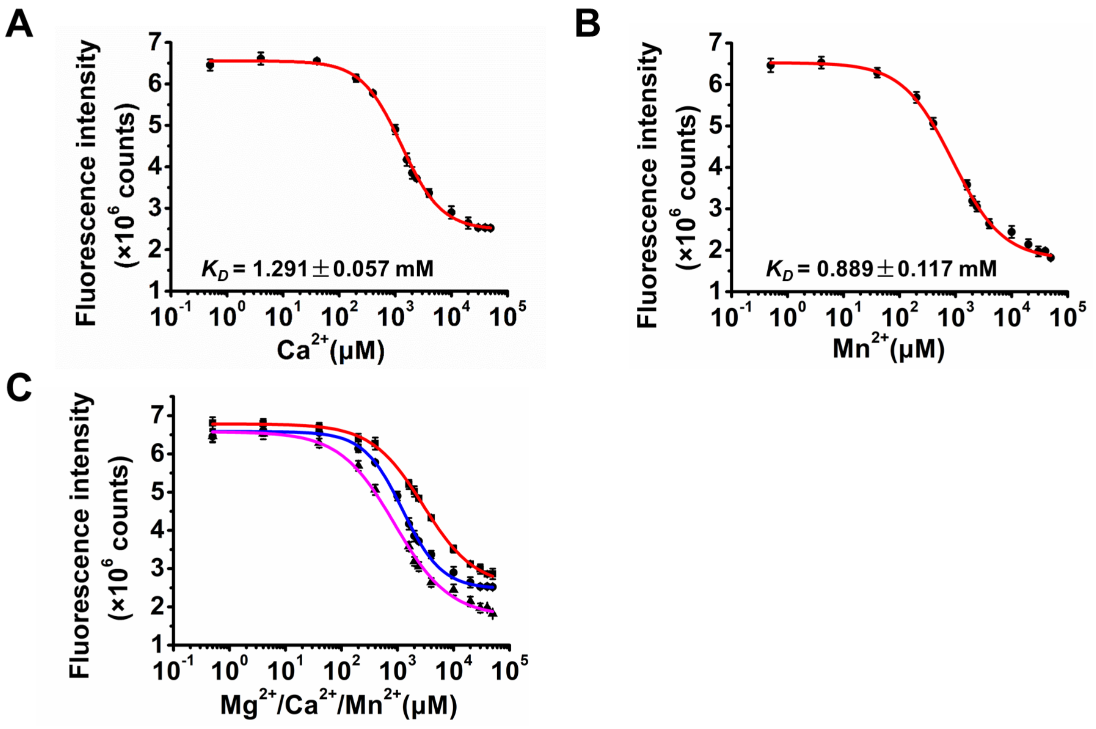

Supplementary Figure S7. Steady-state fluorescence response of TQ3/Cy3-apt to divalent metal ions. (A) Titration curves of 0.5 μM–50 mM Ca^{2+} to TQ3/Cy3-apt. (B) Titration curves of 0.5 μM–50 mM Mn^{2+} to TQ3/Cy3-apt. (C) Superposition of titration curves of Mn^{2+} (magenta), Ca^{2+} (blue) and Mg^{2+} (red) to TQ3/Cy3-apt. Each curve was replicated 3 times, fluorescence intensity values were means ± standard deviations of triplicate experiments.

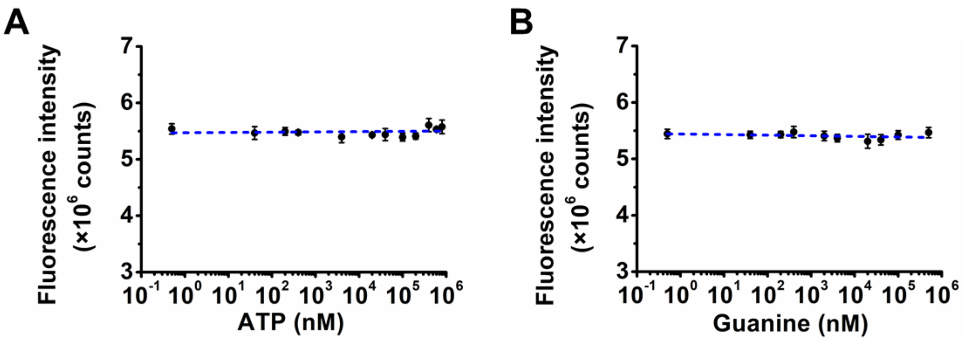

Supplementary Figure S8. Steady-state fluorescence response of TQ3/Cy3-fl to ATP (adenosine triphosphate) and guanine. (A) Titration curves of TQ3/Cy3-fl with the addition of 0.5 nM–1 mM ATP. (A) Titration curves of TQ3/Cy3-fl with the addition of 0.5 nM–1 mM guanine. Each spectrum was replicated 3 times, fluorescence intensity values were means ± standard deviations of triplicate experiments.

Supplementary Table S1. The sequences of primers and DNA templates for adenine riboswitch apt and fl.

Underlined is the T7 promoter sequence. Italicized is the linker between biotin and T7 promoter.

RNA	DNA templates	Sequence
**apt**	non-template strand	5’-biotin-*TCTGATTCAGCTAGTCCA*TAATACGACTCACTATAGGGAAGATATAATCCTAATGATATGGTTTGGGAGTTTCTACCAAGAGCCTTAAACTCTTGATTATCTTCCC
	template strand	5’-mGmGGAAGATAATCAAGAGTTTAAGGCTCTTGGTAGAAACTCCCAAACCATATCATTAGGATTATATCTTCCCTATAGTGAGTCGTATTA*TGGACTAGCTGAATCAGA*
	**Primers for PCR**	**Sequence**
	Forward primer	5’-biotin-TCTGATTCAGCTAGTCCATAATACGACT
	Reverse primer	5’-mGmGGAAGATAATCAAGAGTTTAAGGCTCT
**fl**	**DNA templates**	**Sequence**
	non-template strand	5’-biotin-*TCTGATTCAGCTAGTCCA*TAATACGACTCACTATAGGGAAGATATAATCCTAATGATATGGTTTGGGAGTTTCTACCAAGAGCCTTAAACTCTTGATTATCTTCTCTGTCGCTTTATCCCAAATTTTATAAAGAGAAGACTCATGAAT
	template strand	5’-mAmTTCATGAGTCTTCTCTTTATAAAATTTGGGATAAAGCGACAGAGAAGATAATCAAGAGTTTAAGGCTCTTGGTAGAAACTCCCAAACCATATCATTAGGATTATATCTTCCCTATAGTGAGTCGTATTA*TGGACTAGCTGAATCAGA*
	**Primer for PCR**	**Sequence**
	Forward primer	5’-biotin-TCTGATTCAGCTAGTCCATAATACGACT
	Reverse primer	5’-mAmTTCATGAGTCTTCTCTTTATAAAATTT

Supplementary Table S2. Reagents usage for 2 mL, 15 µM PLOR reaction to generate aa/Cy3-apt and aa/Cy3-fl.

Component/reaction temperature	Concentration
**Initiation stage (Step 1), 37°C, 15min**	
DNA beads	15 µM
T7 RNAP	15 µM
ATP	1.44 mM
GTP	0.96 mM
UTP	144 µM
**Elongation stage (25°C except Step 10, 10min)**	
**Step 2:** ATP,CTP,UTP	30 µM
**Step 3:** ATP, GTP	15 µM
**Step 4:** UTP	15 µM
**Step 5:** ATP, 5-aminoally UTP	15 µM
**Step 6:** ATP,	15 µM
GTP, UTP	90 µM
**Step 7:** ATP, CTP	45 µM
UTP	15 µM
**Step 8:** ATP	15 µM
GTP	30 µM
**Step 9:** CTP, UTP	30 µM
**Step 10 (30°C):**	
ATP	45 µM
Cy3-CTP	15 µM
**Termination stage (25°C for 10min)**	
**Step 11 for aa/Cy3-apt:**	
ATP	30 µM
CTP	75 µM
GTP	15 µM
UTP	120 µM
**Step 11 for aa/Cy3-fl:**	
ATP	225 µM
CTP	105 µM
GTP	90 µM
UTP	210 µM

Supplementary Table S3. K_D values of Mg^{2+} and adenine binding to TQ3/Cy3-apt and TQ3/Cy3-fl.

RNA	Mg^{2+}	Adenine (2 mM Mg^{2+})	Adenine (50 mM Mg^{2+})
**TQ3/Cy3-apt**	2.871 ± 0.050 mM	61.833 ± 0.621 μM	66.546 ± 0.987 μM
**TQ3/Cy3-fl**	3.990 ± 0.078 mM	15.826 ± 0.287 μM	13.229 ± 0.222 μM
